# Predicting Next Day Heart Rate Variability Based on Training Load in Cyclists Using Machine Learning

**DOI:** 10.3390/sports14070271

**Published:** 2026-06-30

**Authors:** Artur Barsumyan, Anton Saukkonen, Christian Soost, Jan Adriaan Graw, Rene Burchard

**Affiliations:** 1 Faculty of Medicine, Philipps-University of Marburg, 35032 Marburg, Germany; 2 Sports Medicine and Joint Centre, Department of Orthopedics and Trauma Surgery, Lahn-Dill-Kliniken, Rotebergstr. 2, 35683 Dillenburg, Germany; 3 Department of Mathematics and Systems Analysis, Aalto University, 02150 Espoo, Finland; 4 Faculty III: Statistic and Econometrics, University of Siegen, 57076 Siegen, Germany; 5 Department of Anaesthesiology, Friedrich-Alexander-Universität Erlangen-Nürnberg (FAU), 91054 Erlangen, Germany; 6 Department of Orthopedics and Traumatology, University Hospital of Giessen and Marburg, 35043 Marburg, Germany

**Keywords:** heart rate variability, training load, machine learning, cycling

## Abstract

Introduction: Day-to-day fluctuations in heart rate variability (HRV) are widely used to infer autonomic recovery in endurance athletes. However, the extent to which HRV can be forecast one day ahead from readily available external and internal training-load metrics remains unclear. In this study, we evaluated whether machine learning models can predict next-day HRV in competitive cyclists using the two load descriptors most commonly collected in practice: external load quantified as total mechanical work in kilojoules (kJ) and internal load quantified as session rating of perceived exertion (RPE). Methods: Seven male competitive endurance cyclists were monitored daily for sixteen weeks, yielding 590 athlete-days of longitudinal data (seven independent time series). Two machine learning approaches—support vector regression (SVR) and extreme gradient boosting (XGBoost)—were compared with a conventional autoregressive model with exogenous inputs (ARX) as a traditional time-series benchmark. Each model was trained individually per athlete under two predictor scenarios (using past HRV-only or past HRV plus kJ and RPE) and across multiple lag orders (1, 4, 7, 10 and 14 days), with forecasting accuracy expressed as root mean squared error (RMSE). Results: Across all athletes, adding kJ and RPE to the past HRV produced only modest reductions in RMSE relative to HRV-only models. XGBoost achieved the lowest one-step-ahead RMSE at short lag, while all models converged at longer lag orders. Predictive accuracy differed markedly between athletes, reflecting the well-known individual nature of autonomic responses. Conclusions: These findings suggest that the two routinely collected load descriptors examined here—total work (kJ) and RPE—add limited information beyond recent HRV history for forecasting next-day HRV, and that broader contextual variables are likely required to meaningfully improve athlete monitoring.

## 1. Introduction

Coaches and athletes now operate in a data-rich environment, where daily metrics offer deeper insight into how athletes respond to and recover from training loads [1]. However, this technological progress has introduced a new challenge: interpreting hundreds of daily data points to guide training decisions [2]. While early performance assessments were largely subjective and revealed discrepancies between the prescribed training stimulus and the stimulus experienced by athletes, modern performance evaluation has evolved into an advanced field of predictive analytics integrated with physiological monitoring [3,4].

Training load regulation is a fundamental cornerstone of performance optimization in competitive sports because it is the main driver of physiological adaptation and long-term performance development [5]. Depending on whether the variables reflect demands imposed on the athlete or the athlete’s physiological response to those demands, training load is commonly divided into external and internal components. In cycling, external load captures the mechanical work performed, such as training volume in hours, power output, and total work in kilojoules (kJ). Internal load can be assessed during and after exercise and reflects the physiological strain elicited by the external stimulus. It is typically indexed by measures such as heart rate, heart rate variability (HRV), blood lactate concentration, oxygen uptake utilization, and rating of perceived exertion (RPE) [6]. Importantly, internal load is not merely a reflection of the mechanical work performed; it also incorporates contextual modifiers, such as environmental heat, accumulated fatigue, sleep quality, and psychosocial stress, that influence the athlete’s stress response to a given external workload [5]. Therefore, monitoring individual responses to training load is essential for designing effective, personalized training programmes [6].

Resting heart rate (RHR) and HRV are valuable non-invasive markers of autonomic recovery and training status [7,8]. They are widely used by athletes and coaches to inform daily training decisions [9]. However, traditional threshold-based interpretations of RHR and HRV can overlook the complex, nonlinear interactions between training load, sleep, recovery dynamics and autonomic function [10]. In particular, functional overreaching can mimic signs of improved fitness in these metrics, making it impossible to differentiate positive adaptation from accumulating fatigue without integrating perceptual measures such as the rating of RPE [11].

In practical coaching settings, particularly in cycling and other endurance disciplines, practitioners must consider various metrics, such as power, training volume, sleep data and wearable-derived recovery indices, as well as subjective reports, for multiple athletes simultaneously. From an applied standpoint, the differences in forecasting accuracy between models were typically below 1–2 ms in rMSSD. This is of the same order as, or smaller than, the night-to-night measurement error of the wearable devices used here (mean absolute errors of roughly 5 ms against ECG references for nocturnal rMSSD) and below the smallest change a practitioner would act upon when assigning an athlete to a “green/amber/red” readiness category. A 1–2 ms reduction in prediction error is therefore better interpreted as a numerical refinement than as a change likely to alter day-to-day coaching decisions. The cognitive and analytical demands of integrating these data sources often exceed human processing capacity, which increases the risk of oversimplified decision-making or delayed detection of maladaptive responses [12]. This highlights a critical methodological gap: the need for analytical frameworks capable of integrating longitudinal, multimodal athlete data at an individual level.

Machine learning (ML) techniques offer a promising approach to address this challenge. Their capacity to model non-linear relationships and accommodate inter-individual variability has motivated a rapidly expanding literature on ML applications in sports science, including performance prediction, injury risk and training response [13,14,15]. Nevertheless, relatively few studies have directly evaluated whether ML methods can forecast short-term autonomic outcomes such as next-day HRV from load metrics routinely recorded in applied settings.

The aim of this exploratory, proof-of-concept study was therefore to evaluate whether next-day HRV in competitive cyclists can be predicted from two routinely collected training-load metrics (kJ and RPE) using machine learning models, and whether their inclusion adds predictive value beyond the information already contained in the athlete’s recent HRV history. Specifically, two machine learning approaches, support vector regression (SVR) and extreme gradient boosting (XGBoost), were compared with a traditional autoregressive model with exogenous inputs (ARX) model under two prediction scenarios: (1) using current HRV-only, and (2) using current HRV together with total work in kJ and RPE. We hypothesized that the inclusion of kJ and RPE would improve prediction accuracy compared with models based solely on current HRV.

## 2. Materials and Methods

### 2.1. Participants and Study Design

Seven male competitive endurance cyclists (mean age 32.6 ± 6.8 years; mean weekly training volume 11.6 ± 2.4 h) were recruited for this study. Inclusion required a minimum training volume of 8 h per week and daily HRV recording with a wearable device. Participants’ data were collected for 16 consecutive weeks, yielding 590 athlete-days of longitudinal data distributed across the seven biologically independent participants. All analyses were performed within each individual athlete, such that each athlete contributed an independent time series rather than independent observations within a pooled dataset. All participants were employed in office-based occupations without night shift work and continued training under their personal coaches. The study was conducted in accordance with the Declaration of Helsinki and approved by the ethics committee of Philipps University of Marburg (25-209 RS, 16 July 2025).

### 2.2. Data Acquisition

For each training session, total mechanical work (kJ) and session RPE (Borg CR10 scale) were recorded within one hour of completion [16]. Nocturnal HRV was measured using either a Garmin wearable (Garmin Inc., Olate, KS, USA) or a WHOOP strap (WHOOP Inc., Boston, MA, USA) and was retained on its native millisecond scale as the root mean square of successive differences between normal R–R intervals (rMSSD) [17,18]. Each athlete used the same wearable device (3 used WHOOP, 4 used Garmin) for the entire 16-week monitoring period. As all models were fitted within athletes, a constant device-specific measurement offset is absorbed into the athlete-specific intercept and baseline. Therefore, there is no bias regarding the within-athlete one-step-ahead forecasts; only relative, within-device fluctuations in HRV inform the models. No participant switched between brands or models during data collection, ensuring within-athlete measurement consistency. We deliberately kept rMSSD untransformed rather than applying the more common natural logarithm transformation to preserve direct comparability with the values displayed in the respective manufacturer applications, which are routinely interpreted by athletes and coaches for applied monitoring purposes. This choice reflects an explicit ecological-validity priority: the training and recovery decisions made by athletes and coaches in real time are driven by the raw rMSSD values displayed by the wearable application, not by an offline natural-logarithm transformation. Modelling the variable on the same scale on which it is actually interpreted aligns the prediction target with its applied use case. All training data were exported from TrainingPeaks (Louisville, CO, USA) and cross-checked in WKO5 (TrainingPeaks, Louisville, CO, USA). Sessions with implausible or corrupted power signals (abrupt spikes, zero-power during active riding, signal loss) were excluded. No interpolation or imputation was performed. Multiple-session days were infrequent and generally consisted of a short low-intensity warm-up or easy ride alongside the main session. As mechanical work is additive, daily kJ was summed across sessions. Because session RPE is not additive, the daily RPE was taken as that of the main (highest intensity) session, avoiding dilution of the day’s principal training stimulus by an ancillary effort. Non-training days were coded as kJ = 0 and RPE = 0. Only athlete-days with a valid next-day HRV value were retained for analysis.

### 2.3. Prediction Targets and Scenarios

The prediction target was next-day HRV (HRV__t+1_). Two predictor scenarios were evaluated for each model: (i) an HRV-only scenario using past HRV values as sole predictors, and (ii) a load-enriched scenario adding daily kJ and RPE as exogenous predictors. Let HRV__t_ denote the rMSSD value recorded on day t. Each scenario was evaluated across five lag orders—1, 4, 7, 10 and 14 days—selected to span the daily to-bi-weekly time scales most relevant to applied HRV monitoring, including the weekly microcycle (7 days) and the 14-day rolling window commonly recommended for HRV-guided training [19], with intermediate values (4 and 10 days) providing additional temporal resolution.

### 2.4. Models and Validation

Separate models were developed for each modelling approach and for each of the two prediction scenarios. For the first scenario, the models were trained using HRV__t_ only. For the second scenario, the models were trained using HRV__t_, kJ and RPE. For each scenario, five candidate lag orders (*p* = {1, 4, 7, 10, 14}) were evaluated using expanding-window, one-step-ahead cross-validation. At each time step t, the model was fitted to all observations up to t and used to predict HRV__t+1_. To ensure comparability across lag orders, a universal start index equal to the maximum lag (*p*_(max) = 14) was applied so that all models produced the same number of predictions over identical test targets.

The ARX model was implemented as:

(1) $$HRV_{t+1}=\;c\;+\;\sum_{k=1}^{p}\varphi_{k}\cdot \;HRV_{t-k+1}+\;\beta_{1}\cdot \;kJ_{t}+\;\beta_{2}\cdot \;RPE_{t}+\;\varepsilon_{t+1},$$

with no differencing (d = 0) and no moving-average component (q = 0). In the HRV-only scenario, the model reduces to a pure AR(*p*). The model was fitted using the statsmodels package [v0.14] via the ARIMA class with order = (*p*, 0, 0). In line with the exploratory, proof-of-concept design of the study, no further hyperparameter optimization was performed beyond lag-order selection through cross-validation; the same baseline-configuration principle was applied to all three model families to ensure that ARX, SVR and XGBoost were compared under directly equivalent specification choices.

SVR was implemented using scikit-learn [v1.7.2] with a radial basis function kernel. The regularization parameter was fixed at C = 100, the ε-insensitive loss margin at ε = 0.1, and the kernel coefficient γ at the scikit-learn default (“scale”, i.e., 1/(n_features × Var(X))). Predictors were standardized to zero mean and unit variance prior to fitting; the standardization parameters were estimated on the training fold only and applied unchanged to the test point at each cross-validation step to avoid information leakage. Hyperparameters were fixed at these values and not tuned, in line with the goal of obtaining a comparable baseline configuration across athletes.

XGBoost was implemented using the xgboost library [v3.2.0] with the following settings: n_estimators = 100, max_depth = 3, learning_rate = 0.1, objective = “reg:squarederror”, random_state = 42. All remaining hyperparameters were kept at the library defaults. As with SVR, predictors were standardized on the training fold and the same transformation was applied to the test point. Hyperparameters were fixed at these values to maintain the equivalent baseline-configuration framework applied to ARX and SVR.

By construction, the ARX HRV-only specification with *p* = 1 is a first-order autoregressive model whose coefficient is estimated from the data, and which nests the persistence forecast HRV_{t + 1} = HRV_t as the constrained special case in which the autoregressive coefficient is fixed at unity. The fitted AR(1) therefore attains an in-sample mean squared error no greater than that of persistence. Analogously, the ARX HRV-only specification with *p* = k generalizes the equal-weight k-day rolling-mean baseline, in which the lag coefficients are constrained to 1/k rather than estimated. Consequently, the lag grid *p* ∈ {1, 4, 7, 10, 14} encompasses, as nested special cases, the trivial forecasting benchmarks most commonly applied in HRV time-series studies—persistence (*p* = 1) and rolling means over weekly (*p* = 7) and bi-weekly (*p* = 14) windows. The ARX HRV-only model is, on these grounds, treated as the naïve baseline against which the augmented ARX (with training-load regressors), SVR, and XGBoost specifications are evaluated.

As reference benchmarks, three naïve forecasts were additionally evaluated under the identical expanding-window, one-step-ahead protocol: persistence (next-day HRV predicted as the most recent observed value) and 7-day and 14-day rolling-mean forecasts. The primary metric for model comparison was the one-step-ahead root mean square error (RMSE), relative performance between models, scenarios and lag orders is interpreted on this scale throughout, and the best lag order per model and scenario was selected as the one minimizing out-of-sample RMSE. To descriptively quantify the consistency of RMSE differences between scenarios, we additionally computed per-step squared-error differences *d__t_* = *e__t_no-exog_ − e__t_exog_* and summarized them using a two-sided Wilcoxon signed-rank test, chosen for its robustness to the right-skewed, heavy-tailed distribution of squared prediction errors. Because these supplementary comparisons share the same underlying RMSE measure, and because the dependence structure inherent to one-step-ahead time-series errors reduces the effective sample size, the resulting *p*-values are reported as descriptive aids supporting the RMSE interpretation rather than as a confirmatory inferential framework; for the same reason, no correction for multiple comparisons was applied. Given the exploratory design and the serial dependence of one-step-ahead errors, the Wilcoxon and paired-t results are reported descriptively and are not interpreted as confirmatory.

All analyses were performed in Python (v3.11).

## 3. Results

A total of 590 athlete-days from seven cyclists were available. Descriptive characteristics and RMSE values for all models, scenarios and lag orders are summarized as best-lag performance per athlete in Table 1. Full per-lag results are provided in Supplementary Table S1.

### 3.1. Overall Model Performance

**Figure 1:**
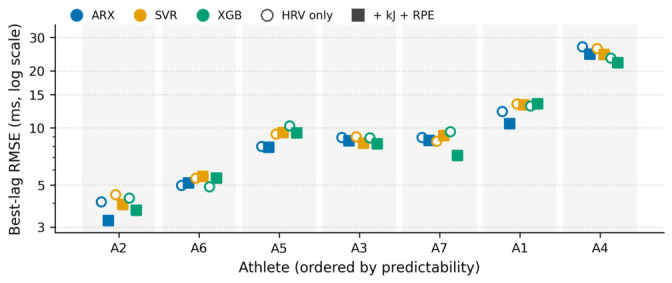
Best-lag RMSE of next-day HRV prediction by athlete, model, and predictor scenario. The RMSE axis (ms) is displayed on a logarithmic scale to accommodate the order-of-magnitude differences in prediction error between athletes.

The three modelling approaches produced broadly comparable one-step-ahead prediction errors across all seven athletes. ARX produced the lowest RMSE for the best lag for most athletes, while XGBoost was the strongest performer for the rest. SVR rarely produced the lowest error in either scenario (Figure 1). Notably, the absolute differences between models for a given athlete were generally small compared to the overall error magnitude, and no consistent ranking emerged across lag orders or athletes. There was more variation in prediction accuracy between athletes than between models, indicating that individual HRV dynamics—driven by factors such as fitness level, training load history and autonomic profile—exerted a stronger influence on forecast quality than the choice of modelling algorithm. Overall, the results suggest that the additional non-linear capacity of the machine learning models offered no systematic advantage over the linear ARX benchmark for the time series lengths and feature set considered here.

### 3.2. Effect of Adding Training-Load Predictors (kJ and RPE)

To determine whether including exogenous variables (kJ and RPE) improved predictive accuracy, we compared one-step-ahead squared prediction errors between scenarios at each time step. For each prediction t, we computed the squared prediction error of each scenario, denoted e__t,no-exog_ for the HRV-only model and e__t,exog_ for the load-enriched model (i.e., e__t_ = (HRV__t_ − ĤRV__t_)^2^). As described in Methods, this comparison is reported as a descriptive complement to the RMSE-based ranking, using a two-sided Wilcoxon signed-rank test applied per lag and to the pooled predictions across all five lag orders.

**Figure 2:**
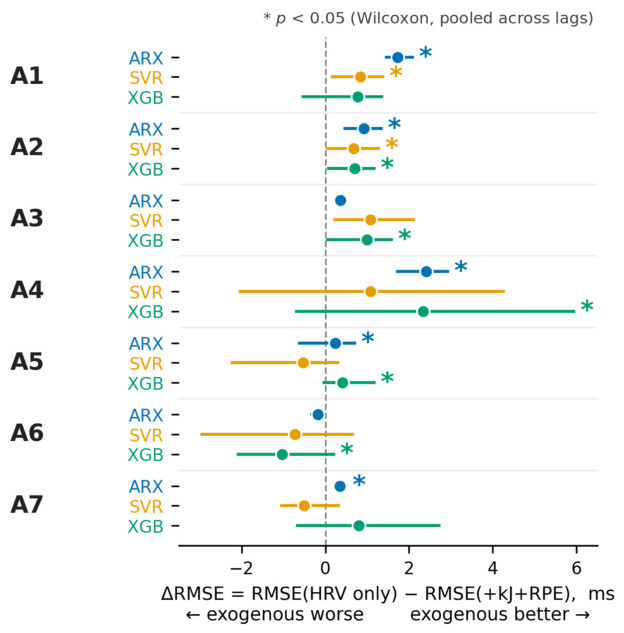
For each athlete and modelling approach, ΔRMSE = RMSE(HRV-only) − RMSE(+kJ + RPE) was computed at each of five lag orders (1, 4, 7, 10 and 14 days). Filled circles show the mean ΔRMSE across lags; whiskers span the minimum and maximum lag-specific value. Positive values denote that exogenous predictors reduced prediction error. Asterisks denote *p* < 0.05 from a two-sided Wilcoxon signed-rank test on per-prediction squared-error differences pooled across all five lag orders.

Across all seven athletes and three modelling approaches, including kJ and RPE as exogenous predictors yielded a lower root mean square error (RMSE) in the majority of athlete–model–lag combinations. In absolute terms these RMSE reductions were small—typically well under 1 ms—and the accompanying significance tests, reported here only as descriptive aids given the autocorrelation of one-step-ahead errors, were inconsistent. When predictions were pooled across all lag orders, the Wilcoxon signed-rank test reached significance (*p* < 0.05) for five of the seven athletes under ARX and XGBoost, but only two of the seven athletes under SVR. At the per-lag level, significance was sporadic; isolated lag–athlete combinations reached *p* < 0.05, but no single lag order was consistently significant across all athletes or models. For instance, athlete 2 demonstrated the most consistent evidence of exogenous benefit, achieving significance at the per-lag level across multiple lags in all three models. In Figure 2, each point shows the mean across-lag change in RMSE for one athlete–model combination, with positive values indicating that adding kJ and RPE reduced prediction error; whiskers span the range across the five lag orders, and asterisks mark pooled-prediction significance. In contrast, athletes 3 and 7 exhibited no significance at the per-lag level under any model (Figure 2).

Taken together, these results indicate that adding kJ and RPE as exogenous predictors consistently reduced one-step-ahead RMSE in most athlete–model–lag combinations, but the magnitude of this reduction was small and the statistical evidence varied across athletes, models and lag orders. The pooled analysis suggests a systematic, albeit modest, benefit of exogenous predictors for the majority of athletes under ARX and XGBoost, while no single lag order emerged as universally informative. The variability of RMSE differences across lag orders and athletes is consistent with a small effect size operating on top of strong individual variability in autonomic responses to training load.

### 3.3. Comparison to Baseline Model

Table 2 reports the naïve baselines. For six of the seven athletes, all fitted models (ARX, SVR and XGBoost) achieved a lower out-of-sample RMSE than naïve persistence, indicating that the models captured more than simple day-to-day carry-over of HRV.

When models were compared by their best-lag RMSE, ARX produced the lowest prediction error for four of the seven athletes under the exogenous scenario and three of the seven under the autoregressive-only scenario. XGBoost performed best for the remaining athletes in both scenarios (three out of seven with exogenous inputs and three out of seven without), while SVR produced the lowest error for only one athlete in one scenario. Despite these differences in ranking, the absolute RMSE gaps between models were generally small. For example, the best-lag errors of the three models often fell within 1–2 ms per minute of each other for the same athlete, and no single machine learning model consistently outperformed the ARX benchmark across participants. These results suggest that for one-step-ahead HRV prediction with the present sample sizes and feature set, the additional flexibility of SVR and XGBoost does not result in systematically greater accuracy than the simpler linear ARX model.

### 3.4. Between-Athlete Variability

The prediction accuracy of athletes varied substantially, with the RMSE of the best predictions differing by roughly an order of magnitude between the most and least predictable individuals. Importantly, the relative ranking of athletes according to prediction difficulty remained largely consistent across all three model families; the same athletes consistently appeared as the easiest or hardest to forecast, regardless of the algorithm used. The between-athlete differences in absolute prediction error were largely a function of each athlete’s HRV scale. Across the cohort, best-model RMSE was strongly correlated with the within-athlete standard deviation of rMSSD (Pearson r = 0.95): athletes with higher absolute HRV and greater day-to-day variability—most notably athlete 4 (mean rMSSD ≈ 117 ms)—showed correspondingly larger absolute errors, whereas athletes with lower, more stable HRV (e.g., athletes 2 and 6) showed the smallest. Because none of these contextual variables were measured here, we can only hypothesize that differences in day-to-day autonomic stability, training regularity and unmeasured lifestyle factors such as sleep quality and psychosocial stress contribute to this variability. The between-athlete differences themselves are the empirical observation; the proposed sources are candidate explanations rather than findings supported by the present data.

## 4. Discussion

To our knowledge, this is the first study of competitive cyclists to compare two ML approaches with a classical autoregressive time series benchmark for forecasting next-day heart rate variability (HRV), under two carefully matched predictor scenarios and across multiple lag orders. Three key findings emerge: the modest incremental value of daily load predictors; the comparatively small gap between ML and the traditional benchmark in terms of physiological significance; and substantial between-athlete heterogeneity in predictive accuracy.

Our first and most significant finding is that adding kJ and RPE to a model with access to the athlete’s recent HRV history only yields small improvements in forecasting accuracy. This contradicts the implicit assumption—prevalent in applied monitoring—that quantifying external and internal load in greater detail should improve recovery prediction [20,21]. From a physiological perspective, HRV on any given morning reflects the net state of a tightly regulated and partially stochastic autonomic system that not only integrates the mechanical work of the previous day, but also sleep quantity and quality, hydration levels, thermoregulatory stress, caffeine and alcohol intake, psychological stress, subclinical illness, and hormonal status [22]. Although daily kJ and RPE are valid markers of cumulative training demand, they capture only a narrow slice of this integrative input [19]. Our results therefore support a more nuanced interpretation of HRV-based recovery monitoring: kJ and RPE remain useful for contextualizing adaptation over longer periods, but, on their own, they are unlikely to serve as sufficient inputs for day-to-day HRV forecasting in the present sample. This conclusion applies specifically to these two metrics of training load and not to training-load monitoring in a broader sense.

A second key finding was that the machine learning models did not demonstrate a systematic accuracy advantage over the linear ARX benchmark. When evaluated using the RMSE at the optimal lag, ARX produced the lowest prediction error for most athletes under both the exogenous and the autoregressive-only scenarios. XGBoost was the strongest performer for the remaining athletes, while SVR rarely achieved the lowest error in either scenario. Importantly, the absolute RMSE differences between the three model families for a given athlete were usually small—often within 1–2 ms—and no consistent ranking emerged across lag orders. This suggests that, given the time series lengths, feature set and baseline parameterisation available in the present study, the additional non-linear capacity of SVR and XGBoost did not translate into a clear forecasting gain over a well-specified linear autoregressive benchmark. The convergence of all three model families on similar prediction errors is consistent with the possibility that next-day HRV forecastability from load-only predictors is constrained primarily by underlying physiology rather than by algorithmic choice, although optimization of model-specific hyperparameters in larger samples may further inform this interpretation.

Third, the accuracy of predictions varied more between athletes than between models. The same individuals consistently proved to be the easiest or hardest to forecast, regardless of the algorithm or scenario used. This suggests that athlete-specific factors are the dominant source of variance in forecast quality rather than method-specific factors. We hypothesize that this pattern reflects stable differences in autonomic regularity, training periodisation structure and unmeasured lifestyle factors such as sleep, psychosocial stress and nutrition. As these were not recorded, they remain candidate explanations to be tested in future work rather than conclusions drawn from the present data. This finding supports the growing consensus that group-level HRV thresholds and normative recommendations are of limited use and that HRV-guided training should be based on individualized reference ranges and trend-based decision rules. Moreover, because every model was estimated separately within each athlete, the present results are most appropriately interpreted as a set of individualized forecasting case studies rather than as evidence of generalizable machine learning performance across athletes.

Taken together, these findings suggest that model sophistication alone is insufficient when the set of predictors is limited. More flexible algorithms appear insufficient, on their own, to compensate for a limited predictor set that may not capture the biological variability underlying day-to-day HRV fluctuations. The next generation of athlete-monitoring tools will likely need to incorporate a broader range of variables—including sleep duration and quality, resting heart rate, recent load history, monotony and strain indices, symptoms of illness, travel and environmental exposure, and indicators of psychosocial stress. In addition, it should explicitly accommodate between-athlete heterogeneity through personalized or hierarchical modelling.

### Limitations

These results should be interpreted in the context of several limitations. First, the cohort consisted of seven male endurance cyclists, and the study is therefore best characterized as an exploratory, proof-of-concept investigation rather than a population-level test of HRV forecastability. Second, for long lag orders and short effective training series, the observed RMSE values, particularly for SVR, should be interpreted with caution, as they may reflect modelling flexibility on autocorrelated data rather than generalizable predictive performance. Third, HRV was recorded using consumer wearables, both of which derive nocturnal rMSSD from wrist-based photoplethysmography and have been validated for HRV monitoring in applied settings [18]. Because each athlete used a single device consistently throughout the entire 16-week monitoring period, and because all models were fitted and evaluated within an individual athlete, the inferential framework is by design insensitive to between-brand differences in absolute rMSSD scaling. The reported per-athlete RMSE values therefore reflect within-device, within-athlete forecasting accuracy. Furthermore, rMSSD was modelled on its native millisecond scale rather than after the natural-logarithm transformation sometimes applied in HRV time-series analysis. This was a deliberate methodological choice driven by ecological validity: the prediction target was aligned with the form in which HRV is presented to athletes and coaches in daily monitoring. Although raw rMSSD is known to be right-skewed and heteroscedastic, the inferential framework used here is robust to these features by design—all models were fitted within individual athletes, eliminating the influence of cross-subject scale heterogeneity on the within-athlete RMSE that constitutes our primary outcome, and statistical comparisons of squared errors were conducted using the rank-based Wilcoxon signed-rank test, which does not rely on Gaussian residual assumptions. Future studies with larger cohorts may complement this applied parameterisation with a log-transformed analysis to provide a parallel statistical perspective. We further acknowledge that, because much of the existing HRV literature reports ln(rMSSD), our use of the untransformed scale may limit direct numerical comparability with those studies. Fourth, the predictor set was intentionally restricted to two load metrics routinely collected in practice. While this maximizes ecological relevance, it excludes other known determinants of HRV—including sleep duration and quality, resting heart rate, hydration, psychological stress, illness, and environmental exposure—that are likely necessary to capture the integrative physiological input shaping autonomic recovery. Fifth, all three model families were evaluated under fixed baseline configurations rather than a formal hyperparameter-optimization procedure. This choice was made deliberately to keep ARX, SVR and XGBoost on directly comparable specification footing within the exploratory scope of the study.

## 5. Conclusions

These results should be regarded as preliminary, hypothesis-generating evidence from seven individual athletes rather than as generalizable findings, particularly given the marked variability in forecasting accuracy observed between athletes.

Over a period of sixteen weeks, daily HRV and training-load data were collected from seven competitive cyclists. Machine learning models (SVR, XGBoost) and a classical autoregressive benchmark (ARX) produced broadly comparable one-step-ahead prediction error for next-day HRV, with no model showing a consistent advantage across athletes. The classical ARX benchmark achieved the lowest prediction error in most cases, while XGBoost was the strongest performer for the remainder. However, the advantage was small in physiological terms and diminished at longer lag orders. Adding external (kJ) and internal (RPE) load data to the athletes’ HRV histories only produced modest reductions in prediction error, and substantial between-athlete heterogeneity was observed in forecasting accuracy. Because models were developed independently for each athlete, these findings should be read as individualized, exploratory case studies rather than as generalizable machine learning benchmarks.

In this exploratory cohort, the two daily training load metrics examined (kJ and RPE) did not, on their own, yield meaningful improvements in next-day HRV forecasts, and more sophisticated ML methods did not compensate for this limitation under the baseline configurations evaluated here. Improving the prediction of individualized recovery in endurance sports will likely require richer contextual inputs and modelling frameworks that explicitly recognize the individual nature of the autonomic response to training.

## Figures and Tables

**Table 1:** Best-lag performance per athlete. For each athlete, model and predictor scenario, the minimum out-of-sample RMSE (ms) across the five evaluated lag orders is reported, with the corresponding lag order in parentheses.

Athlete	ARX (+kJ + RPE)	ARX (HRV-only)	SVR (+kJ + RPE)	SVR (HRV-Only)	XGB (+kJ + RPE)	XGB (HRV-Only)
1	10.54 (4)	12.21 (7)	13.24 (14)	13.40 (14)	13.41 (7)	13.08 (1)
2	3.24 (7)	4.07 (7)	3.94 (14)	4.44 (14)	3.67 (4)	4.27 (14)
3	8.54 (1)	8.93 (14)	8.31 (1)	8.99 (1)	8.23 (10)	8.89 (10)
4	24.54 (7)	26.83 (7)	24.41 (4)	26.29 (14)	22.09 (4)	23.38 (4)
5	7.90 (14)	8.01 (7)	9.43 (1)	9.31 (1)	9.41 (1)	10.26 (10)
6	5.21 (10)	4.96 (7)	5.54 (14)	5.42 (14)	5.44 (14)	4.89 (7)
7	8.6 (14)	8.92 (14)	9.09 (10)	8.50 (10)	7.141 (1)	9.56 (7)

**Table 2:** Out-of-sample RMSE (ms) of naïve baseline forecasts per athlete, computed under the same expanding-window, one-step-ahead protocol used for the models in Table 1.

Athlete	Persistence	7-Day Rolling Mean	14-Day Rolling Mean	ARX (HRV-Only)	Best Model
1	16.29	12.93	12.58	12.21	10.54
2	5.83	4.27	4.21	4.07	3.24
3	10.29	8.64	8.47	8.93	8.23
4	39.00	25.71	24.61	26.83	22.09
5	9.06	10.32	11.17	8.01	7.90
6	3.92	4.03	4.95	4.97	4.89
7	11.54	10.17	10.34	8.92	7.14

## Data Availability

The raw athlete data cannot be shared publicly owing to participant privacy. The analysis code (provided as a link to a repository) is available from the corresponding author upon reasonable request.

## Supplementary Materials

The following supporting information can be downloaded at: https://www.mdpi.com/article/10.3390/sports14070271/s1, Table S1: Per-athlete RMSE (ms) for each model, scenario and lag order.

## Abbreviations

The following abbreviations are used in this manuscript:

ARX	autoregressive model with exogenous inputs
HRV	heart rate variability
rMSSD	the root mean square of successive differences
ML	machine learning
RHR	Resting heart rate
RMSE	root mean squared error
RPE	rating of perceived exertion
SVR	support vector regression
XGB	extreme gradient boosting
