# Supplementary material for: Predicting Next Day Heart Rate Variability Based on Training Load in Cyclists Using Machine Learning
Source: Sports (Basel). 2026 Jun 30;14(7):271. doi: 10.3390/sports14070271 (PMC13418909; doi:10.3390/sports14070271)
Supplement: Supplementary file 1 [file sports-14-00271-s001.zip › sports-4356656-supplementary.pdf]

Table 1. Per-athlete RMSE (ms) for each model, scenario and lag order.

Athlete	Model	Scenario	lag=1	lag=4	lag=7	lag=10	lag=14
**1**	ARX	+kJ+RPE	11.03	10.54	10.74	10.69	10.75
	ARX	HRV only	13.11	12.54	12.21	12.32	12.21
	SVR	+kJ+RPE	13.79	16.69	15.35	13.56	13.24
	SVR	HRV only	14.01	17.92	16.57	14.93	13.40
	XGB	+kJ+RPE	13.61	13.87	13.41	13.50	13.81
	XGB	HRV only	13.08	15.22	14.39	14.61	14.78
**2**	ARX	+kJ+RPE	3.44	3.42	3.24	3.46	3.85
	ARX	HRV only	4.71	4.75	4.07	4.15	4.32
	SVR	+kJ+RPE	4.65	4.99	4.71	4.01	3.94
	SVR	HRV only	4.71	5.6	5.98	4.98	4.44
	XGB	+kJ+RPE	4.1	3.67	3.86	4.2	3.88
	XGB	HRV only	5.02	4.83	4.79	4.28	4.27
**3**	ARX	+kJ+RPE	8.54	8.63	9.28	8.72	8.67
	ARX	HRV only	8.95	9.1	9.59	9.06	8.93
	SVR	+kJ+RPE	8.31	9.15	9.26	8.90	8.4
	SVR	HRV only	8.99	10.64	10.15	9.12	10.50
	XGB	+kJ+RPE	8.7	9.66	8.84	8.23	8.84
	XGB	HRV only	10.14	11.24	10.07	8.89	8.90
**4**	ARX	+kJ+RPE	27.42	25.36	24.54	24.97	25.02
	ARX	HRV only	29.14	27.85	26.83	27.62	27.94
	SVR	+kJ+RPE	30.91	24.41	25.03	25.25	26.49
	SVR	HRV only	28.88	26.46	29.28	26.55	26.29
	XGB	+kJ+RPE	25.32	22.09	23.63	23.96	27.88
	XGB	HRV only	31.25	23.38	26.36	26.40	27.19
**5**	ARX	+kJ+RPE	8.31	8.71	8.63	8.38	7.90

	ARX	HRV only	8.69	8.99	8.01	8.83	8.6
	SVR	+kJ+RPE	9.43	11.60	12.08	12.19	11.96
	SVR	HRV only	9.31	10.80	9.85	12.49	12.14
	XGB	+kJ+RPE	9.41	10.73	10.23	10.29	11.70
	XGB	HRV only	10.57	10.81	10.58	10.26	12.16
**6**	ARX	+kJ+RPE	5.33	5.32	5.29	5.12	5.15
	ARX	HRV only	5.13	5.09	4.97	5.00	5.13
	SVR	+kJ+RPE	8.62	7.39	7.05	6.31	5.54
	SVR	HRV only	5.67	6.77	7.70	5.71	5.42
	XGB	+kJ+RPE	7.53	6.93	5.93	5.70	5.44
	XGB	HRV only	5.45	5.26	4.89	5.14	5.64
**7**	ARX	+kJ+RPE	9.01	9.01	8.92	8.71	8.60
	ARX	HRV only	9.50	9.39	9.21	8.99	8.92
	SVR	+kJ+RPE	9.43	12.23	10.36	9.09	9.32
	SVR	HRV only	9.74	11.18	9.49	8.50	8.99
	XGB	+kJ+RPE	7.14	8.49	9.26	10.39	9.44
	XGB	HRV only	9.85	9.79	9.56	9.72	9.78
